# Green Extraction of Reed Lignin: The Effect of the Deep Eutectic Solvent Composition on the UV-Shielding and Antioxidant Properties of Lignin

**DOI:** 10.3390/ijms25158277

**Published:** 2024-07-29

**Authors:** Olga Morozova, Irina Vasil’eva, Galina Shumakovich, Maria Khlupova, Vyacheslav Chertkov, Alla Shestakova, Alexander Yaropolov

**Affiliations:** 1 A. N. Bach Institute of Biochemistry, Research Center of Biotechnology of the Russian Academy of Sciences, Leninsky Ave. 33, 119071 Moscow, Russia; morozova@inbi.ras.ru (O.M.); ir-vas@yandex.ru (I.V.); shumakovich1945@yandex.ru (G.S.); dave80@yandex.ru (M.K.); 2 Department of Chemistry, Lomonosov Moscow State University, Leninskie Gory 1/3, 119991 Moscow, Russia; vchertkov@hotmail.com; 3 State Research Institute of Chemistry and Technology of Organoelement Compounds, Shosse Entuziastov 38, 111123 Moscow, Russia; alshestakova@yandex.ru

**Keywords:** deep eutectic solvent, reed lignin, antioxidant activity, lignin/polyvinyl alcohol composite films, UV shielding

## Abstract

Lignin, the second most abundant natural polymer, is a by-product of the biorefinery and pulp and paper industries. This study was undertaken to evaluate the properties and estimate the prospects of using lignin as a by-product of the pretreatment of common reed straw (*Phragmites australis*) with deep eutectic solvents (DESs) of various compositions: choline chloride/oxalic acid (ChCl/OA), choline chloride/lactic acid (ChCl/LA), and choline chloride/monoethanol amine (ChCl/EA). The lignin samples, hereinafter referred to as Lig-OA, Lig-LA, and Lig-EA, were obtained as by-products after optimizing the conditions of reed straw pretreatment with DESs in order to improve the efficiency of subsequent enzymatic hydrolysis. The lignin was studied using gel penetration chromatography, UV-vis, ATR-FTIR, and ^1^H and ^13^C NMR spectroscopy; its antioxidant activity was assessed, and the UV-shielding properties of lignin/polyvinyl alcohol composite films were estimated. The DES composition had a significant impact on the structure and properties of the extracted lignin. The lignin’s ability to scavenge ABTS^+•^ and DPPH^•^ radicals, as well as the efficiency of UV radiation shielding, decreased as follows: Lig-OA > Lig-LA > Lig-EA. The PVA/Lig-OA and PVA/Lig-LA films with a lignin content of 4% of the weight of PVA block UV radiation in the UVA range by 96% and 87%, respectively, and completely block UV radiation in the UVB range.

## 1. Introduction

Lignocellulose is a valuable natural, renewable raw material. It can replace fossil resources, such as natural gas, oil, and coal. This biomass can be used to produce a vast number of products, such as biofuels, and various chemical compounds and materials [1,2,3]. Lignocellulose primarily consists of three main components, namely, cellulose, hemicellulose, and lignin, each of which is important for further processing into useful products. 

Lignin is the second most abundant biopolymer after cellulose, and it is a valuable natural resource [4]. It has been estimated that approximately 50 million tons of lignin can be available from the annual processing of pulp and paper manufacture waste, but only 2% of it is processed [5]. The availability, biodegradability, and appropriate mechanical properties of lignin make it an attractive object for practical use in different fields [6,7,8,9]. 

Lignocellulose processing encourages the attention of various research groups. Conventional pretreatment processes for this renewable raw material using inorganic acids, alkalis, and organic solvents are environmentally unfriendly, require corrosion-resistant equipment, and often proceed at increased temperatures and under high pressure. Ionic liquids are promising solvents for the fractionation of lignin from lignocellulose, but their wide industrial use is limited by the complexity of their synthesis, high cost, toxicity, and poor biodegradability [10,11]. In contrast to the conventional methods, lignocellulose pretreatment with deep eutectic solvents (DESs) is very promising and meets the requirements of sustainable chemistry [10,11,12,13,14]. Typically, DESs are obtained by the thermal mixing of two components (a hydrogen bond acceptor and a hydrogen bond donor), which are environmentally friendly low-cost compounds [15,16,17]. DESs are characterized by low volatility, conductivity, and non-toxicity. Most of them have high thermal stability, and many of them are biodegradable. The properties of DESs depend on the nature and ratio of their components. As was shown in [18], the physicochemical characteristics of choline chloride-based DESs are mainly dependent on the nature of the hydrogen bond donor. It is important to note that DESs are environmentally friendly solvents and can be efficiently used to extract poorly water-soluble biologically active compounds [19].

Lignin, unlike cellulose, is highly soluble in many DESs. A number of studies aimed to assess the potential of using DESs for the removal of lignin from biomass, and examined the solubility of technical lignins in DESs of various compositions [20,21,22]. The composition, structure and, accordingly, properties of lignin depend on both the original lignocellulosic material and the extraction method [11,23,24,25,26]. In general, lignins have antioxidant and antimicrobial properties, UV-shielding capacity, biocompatibility, and low cytotoxicity. Therefore, they can be converted into valuable products such as hydrogels, packaging materials, sunscreens, UV-shielding materials, etc. [27,28,29,30,31,32,33]. In addition, the use of lignin in high-value-added products is also important for the economic efficiency of biorefineries [34,35]. 

We have shown earlier that after optimizing the pretreatment of reed straw with DESs of different compositions based on choline chloride as a hydrogen bond acceptor, a high efficiency of enzymatic hydrolysis of the cellulose substrate was achieved [36,37], and lignins were obtained as by-products. The purpose of this work was to study the structure and properties of lignins extracted from reed straw using DESs in order to evaluate their antioxidant activity, as well as the UV-shielding capabilities of lignin/polyvinyl alcohol films.

## 2. Results and Discussion

Three lignin samples (Lig-OA, Lig-LA, and Lg-EA) were obtained as by-products after optimizing the conditions of reed straw pretreatment with choline chloride-based DESs. The extracted lignins and cellulosic substrates varied in color (Supplementary Figure S1). The yield of lignin and cellulose substrate was 8.2, 7.0, and 6.1 and 44.3, 56.7, and 47.1 wt.% of the weight of the original reed straw, after pretreatment with ChCl/OA, ChCl/LA, and ChCl/EA, respectively. Due to the complex bonding between lignins and carbohydrates, the extracted lignin usually contained some sugars [38]. The content of polysaccharides in the lignin samples was determined using HPLC as insignificant (3.4–3.9 wt.%); thus, these lignin samples can be suitable for future applications without additional treatment. All three lignin samples were completely dissolved in dimethylsulfoxide (DMSO) and *N*-methyl-2-pyrrolidone. The weight-average molecular weights and polydispersity indices of acetylated Lig-OA, Lig-LA, and Lig-EA were 3800, 5600, and 6400 g mol^−1^, and 1.58, 1.81, and 1.94, respectively.

### 2.1. Spectral Properties of Lignins 

**Figure 1:**
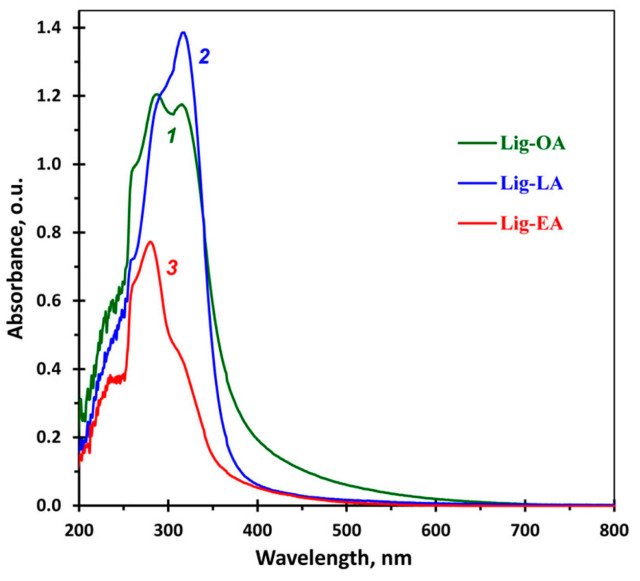
UV–visible spectra of Lig-OA (*1*), Lig-LA (*2*), and Lig-EA (*3*). The lignin concentration is 50 μg mL^−1^.

Figure 1 shows the UV–visible spectra of lignins in DMSO. As can be seen, the shapes of the spectra in the UV region significantly varied depending on the DES composition. The shape of a lignin spectrum is known to depend on many factors, such as the lignin source, extraction method, solvent, etc. The spectra of lignins have a typical absorption peak at 280–282 nm, whose intensity depends on both the ratio of the various lignin structural units (*p*-hydroxyphenylpropan (H), guaiacyl (G), and syringyl (S)) and the extinction coefficient of each unit. The extinction coefficient of the G units at 280 nm is 3.5 times as high as that of the S units; the extinction coefficient of the H units is lower than that of the G units, but higher than that of the S units [39]. Therefore, high absorption in the region of 280 nm could be associated with a relatively high proportion of G units [40]. In addition, grass lignins have a maximum or a shoulder in the region of 315 nm. According to the literature, the absorption in this region can be associated with the n → π* transition in the lignin units, containing C_α_=O groups, and the π → π* transition in the lignin units with C_α_=C_β_ bonds conjugated to the aromatic ring [40], as well as with the esters of *p*-coumaric and ferulic acids present in the lignin structure [39,41]. The spectrum of Lig-OA contains two maxima of almost identical intensity at 286 nm and 317 nm. The Lig-LA spectrum shows an intense maximum at 317 nm and a shoulder at 286 nm. In the Lig-EA spectrum, the intensity of both peaks is significantly lower as compared to Lig-OA and Lig-LA at the same concentration, which may result from the hydrolysis of ester bonds during the treatment of reed straw with alkaline DES (ChCl/EA). Such differences in the spectra of lignin samples suggest that the DES composition has a significant impact on the lignin structure.

**Figure 2:**
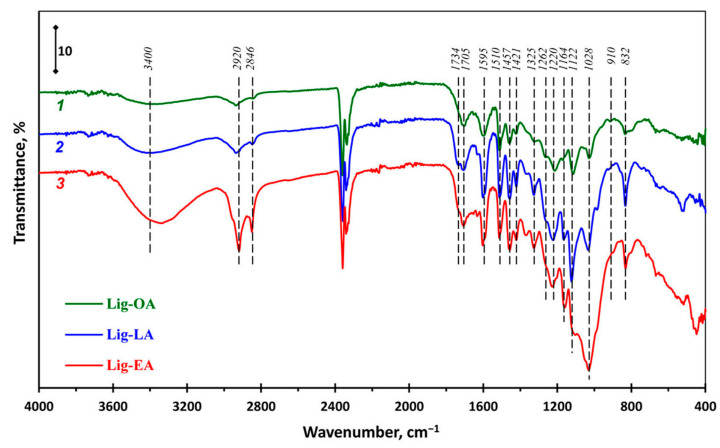
The ATR-FTIR spectra of Lig-OA (*1*), Lig-LA (*2*), and Lig-EA (*3*).

In order to study the structural characteristics of lignins, ATR-FTIR spectra of lignin samples were recorded (Figure 2). The results showed that the spectra of the three lignin samples were almost identical and were generally consistent with typical lignin spectra [40,42,43,44]. Lig-OA and Lig-LA had a broad band at 3400 cm^−1^ attributed to OH groups in phenolic and aliphatic structures. In the case of Lig-EA, this band was shifted to short wavelengths (3330 cm^−1^). All the spectra contained bands at around 2920 cm^−1^ and 2846 cm^−1^, which resulted from the asymmetrical and symmetrical stretching vibrations of the C−H bond in the methyl and methylene groups. The highest intensity of these bands was observed in Lig-EA. The carbonyl/carboxyl region of the spectra demonstrated bands at 1700–1740 cm^−1^ originating from unconjugated carbonyl/carboxyl stretching. At the same time, there was no signal at 1734 cm^−1^ in Lig-OA. Aromatic skeleton vibrations at 1421, 1510, and 1595 cm^−1^ and asymmetric C_ar_–H deformations at 1457 cm^−1^ were common to all three lignins, although the intensity of these bands was different. This indicates that the benzene ring skeleton of the lignin samples was not destroyed during DES pretreatment. The spectral region below 1400 cm^−1^ is more difficult to analyze, since most bands are complex, with contributions from various vibration modes. However, this region contains vibrations specific to different lignin units, which enables a comparison of the structure of the lignin samples. In the spectra of all three lignins, vibrations of different intensities characteristic of the G (1028, 1220, 1262 cm^−1^), S (1325 and 1122 cm^−1^), and H units (1164 cm^−1^) were observed [40,43,45,46,47]. It should be noted that the influence of carbohydrate impurities (1000–1300 cm^−1^) is the most substantial in the Lig-EA spectrum. The band at 832 cm^−1^, which is especially intense in Lig-LA, and a small peak at 910 cm^−1^, can be attributed to the C–H out-of-plane deformation in positions 2, 6 and 2, 5, 6 of the aromatic ring [40,46].

Supplementary Figure S2 shows the ATR-FTIR spectra of cellulosic substrates after reed straw pretreatment with various DESs. The broad maximum in the region of 3400 cm^−1^ is associated with vibrations of the OH groups of lignin and carbohydrates (cellulose and hemicellulose) [48]. The band at around 898 cm^−1^ representing the *β-*(1,4)–glycosidic bond in cellulose [49] becomes more pronounced after pretreatment with DESs. Vibrations at 1034, 1055, and 1105 cm^−1^ related to the stretching vibration of the C–O–C bonds in carbohydrates [49,50] decrease, which indirectly indicates the partial degradation of carbohydrates during the pretreatment. There were no peaks corresponding to aromatic skeleton vibrations and C_ar_–H deformations in the spectra of cellulose substrates after reed straw pretreatment with ChCl/OA and ChCl/EA, which was indicative of a better removal of lignin from reeds as compared to ChCl/LA.

**Figure 3:**
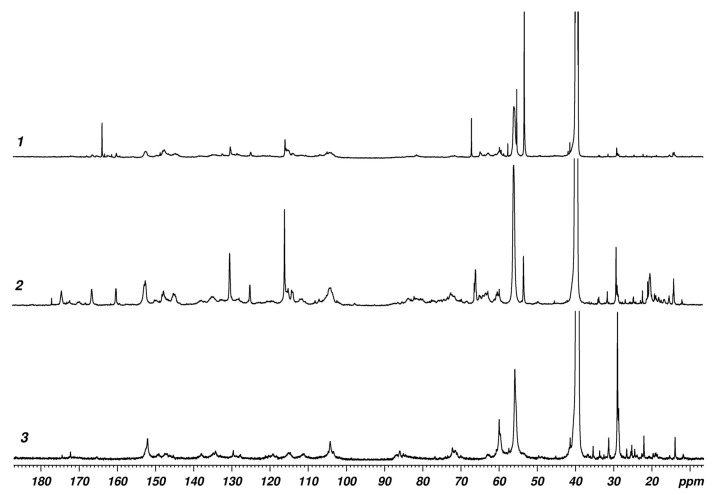
^13^C NMR spectra of Lig-OA (*1*), Lig-LA (*2*), and Lig-EA (*3*).

NMR spectroscopy was also used to estimate the effect of DES composition on the structure of the extracted lignin. It should be noted that it is rather difficult to analyze ^1^H NMR spectra of lignin due to the great variety of bonds between lignin units. ^13^C NMR spectroscopy is a more effective and informative method for studying lignin. In the ^13^C NMR spectra of lignin, signals from more than 40 types of carbon atoms can be distinguished, which, based on chemical shifts, are divided into segments of carbonyl groups (160–185 ppm), benzene rings and side chain double bonds (100–160 ppm), and side chains and methoxy groups (10–100 ppm) [51]. Figure 3 shows the ^13^C NMR spectra of Lig-OA, Lig-LA, and Lig-EA. The literature data on chemical shifts of ^13^C from monographs [52] was used to assign the signals in the spectra with structural fragments characteristic of lignin. For a more reliable assignment of the ^13^C signals, J-modulated spin-echo experiments were also performed [53].

All three lignin samples studied showed significant differences in the ^13^C NMR spectra. Lig-LA had the greatest structural diversity; it showed many signals in the region of carbonyl groups (160–185 ppm), aromatic carbon atoms (110–155 ppm), and aliphatic carbon atoms bound to oxygen (70–80 ppm). The spectrum of Lig-OA also has many signals in the aromatic region, but there is no such diversity in the region of 70–80 ppm; the carbonyl region revealed one signal (162 ppm) characteristic of oxalic acid. It should be noted that the Lig-LA spectrum contained signals characteristic of lactic acid (21, 66, 176 ppm), which indicates possible lignin modification with acids during the pretreatment of reed straw with ChCl/OA and ChCl/LA. Also, both spectra demonstrated typical signals of methoxy groups attached to aromatic (55 ppm) and aliphatic (53 ppm) carbon atoms. The Lig-EA spectrum contained a variety of carbon signals in the region of aromatic structures (110–155 ppm) and aliphatic carbons bound to oxygen (70–80 ppm), but there were virtually no peaks in the carbonyl region (160–185 ppm). The ^13^C NMR spectrum of this lignin had no signal characteristic of the methoxy groups attached to aliphatic carbon atoms. An intense signal in the region of 29 ppm in the Lig-EA spectrum, characteristic of CH_2_ groups, may be associated with the reduction of the double bond in the side chains of lignin with monoethanolamine during the pretreatment of reed straw with ChCl/EA.

Comparative analysis of the ^13^C NMR spectra of the lignin samples investigated in this work allows us to conclude that the greatest structural diversity is observed in Lig-LA. This lignin contains numerous carbonyl groups and oxygen-linked CH_2_ groups (−O−CH_2_−R). In contrast, Lig-OA and Lig-EA do not contain significant signals in the region of carbonyl groups. In addition, the Lig-EA spectrum contains only signals characteristic of methoxy groups attached to aromatic carbon atoms.

Thus, the spectroscopic studies showed that all three DESs effectively removed lignin from reed straw, and the structure of the extracted lignin was dependent on the DES composition.

### 2.2. Antioxidant Activity and the Total Phenolic Content of Lignins

The antioxidant activity of lignin is directly related to its structure, so structural changes in lignin inevitably cause changes in its biological activity. Phenolic hydroxyl groups as well as conjugated double bonds in lignin have a positive effect on the antioxidant activity, while aliphatic hydroxyl groups of lignin negatively correlate with the antioxidant activity [54,55,56].

**Figure 4:**
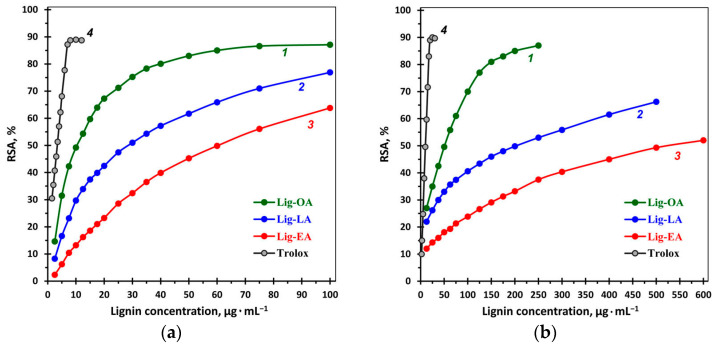
ABTS^•+^ (**a**) and DPPH^•^ (**b**) radical scavenging activity of Lig-OA (*1*), Lig-LA (*2*), and Lig-EA (*3*) in comparison to Trolox (*4*).

In order to evaluate the antioxidant potential of lignins, ABTS^+•^ and DPPH^•^ were used, which are the most widely applied and stable chromogenic radicals for measuring the antioxidant activity of biological materials. The commercial antioxidant Trolox was used as a positive control. As shown in Figure 4, the radical scavenging capacity of all three lignins rose with an increasing lignin concentration, and in both ABTS^+•^ and DPPH^•^ assays, the antioxidant activity decreased as follows: Lig-OA > Lig-LA > Lig-EA (Table 1). It is noteworthy that the ABTS^+•^ free radical scavenging ability of Lig-OA was not much lower (IC_50_ = 10 μg mL^−1^) than that of Trolox (IC_50_ = 3 μg mL^−1^). The removal of the ABTS^+•^ radical by all three lignins was much higher than that of the DPPH^•^ radical. These differences are related to the mechanism of the reactions; the ABTS^+•^ radical reactions involve electron transfer and occur at a much higher rate compared to DPPH^•^ radicals [57]. Similar results were obtained by other researchers. For example, the IC_50_ values for BIOLIGNIN™ (lignin extracted from wheat straw) were 0.04–0.05 mg mL^−1^ in the DPPH assay and 0.009–0.01 mg mL^−1^ in the ABTS assay [58]; for lignin extracted by various methods from corncob, 0.17–0.26 mg mL^−1^ (DPPH^•^) and 0.016–0.028 mg mL^−1^ (ABTS^+•^) [59]; for industrial kraft and organosolv lignins, 3.47–5.16 μg mL^−1^ (ABTS^+•^) and 12.85–22.75 μg mL^−1^ (DPPH^•^) [60]. Tavares et al. reported an IC_50_ value of 60 μg mL^−1^ in the DPPH assay and 7.39 μg mL^−1^ in the ABTS assay for lignin extracted from *Eucalyptus* spp. sawdust [61], and Wei et. al. reported IC_50_ values of 262.87–1704.38 mg L^−1^ (ABTS^+•^) and 267.47–1730.82 mg L^−1^ (DPPH^•^) for lignin fractions obtained from eucalyptus kraft lignin by solvent extraction [62]. The differences in the antioxidant activity of lignin samples are related to the extraction methods, as well as to the source of lignocellulosic material [63].

Our study demonstrated a correlation between the content of phenolic groups and the antioxidant activity of the lignin samples (Table 1). Out of the three lignin samples, Lig-OA showed the highest total phenolic content (TPC ~170 mg GAE g^−1^), and the highest IC_50_ value (10 and 50 μg mL^−1^ in ABTS^•+^ and DPPH^•^ assays), while Lig-EA showed the lowest antioxidant activity and the lowest content of phenolic groups.

The antioxidant properties of nanosized lignin particles (NPLig) obtained from original lignins by solvent exchange via dialysis were also studied [64]. Nanosized lignin arouses great interest because its large specific surface area has a significant impact on its physicochemical properties [65,66]. The resulting nanosized lignin particles (NPLig-OA, NPLig-LA, and NPLig-EA) had average sizes of 160, 200, and 250 nm and zeta potentials of −24.19, −31.41, and −24.78 mV, respectively. As in the case of the original lignins, the ABTS^+•^ radical scavenging ability of all three nanosized lignins rose with increases in the NPLig concentration (Supplementary Figure S3), while the antioxidant activity decreased as follows: NPLig-OA > NPLig-LA > NPLig-EA (IC_50_~10, 25, and 55 μg mL^−1^). Thus, no significant differences in the antioxidant activity of nanosized lignin particles and original lignins were observed.

The results obtained confirm the fact that the composition of the DES used for reed straw pretreatment has a significant effect on the structure and, consequently, the antioxidant properties of lignin.

### 2.3. Optical and UV-Protective Properties of Polyvinyl Alcohol/Lignin Films

The phenolic structures of lignin are known to have excellent UV-shielding properties [67]. Therefore, in many studies, lignin is used as a component of biodegradable films based on various polysaccharides, poly(lactic acid), PVA, and other polymers [67,68,69,70,71,72].

**Figure 5:**
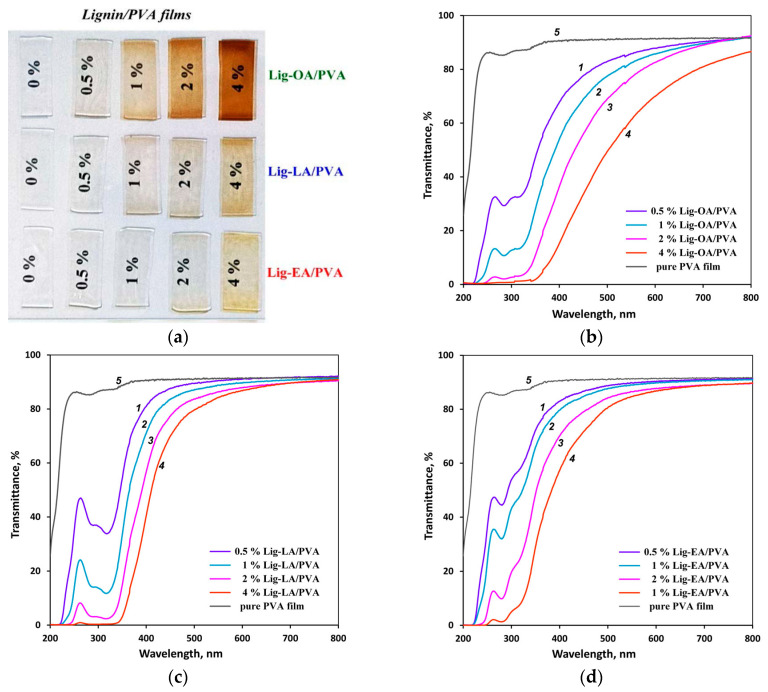
Digital photographs (**a**) and UV-vis light transmittance spectra of the PVA/Lig-OA (**b**), PVA/Lig-LA (**c**), and PVA/Lig-EA (**d**) films with different lignin contents (0.5% (*1*), 1% (*2*), 2% (*3*), 4% (*4*)) in comparison to the pure PVA film (*5*).

In order to evaluate the UV-shielding ability of Lig/PVA films, the light transmittance of all the samples was measured in the wavelength range of 250–800 nm. Figure 5 shows photographs and UV-vis transmittance spectra of pure PVA films and PVA composite films with different lignin contents. The thickness of all the films was about 60 μm.

The pure PVA film transmitted 90% of ultraviolet radiation in the range of 200–400 nm. As shown in Figure 5, the inclusion of lignin in the PVA matrix significantly shields UV radiation, and the shielding efficiency increases as follows: Lig-OA > Lig-LA > Lig-EA. For all three lignins, the increase in the lignin content from 0.5 to 4% assured higher UV protection. In general, the PVA/Lig-OA and PVA/Lig-LA films showed a higher UV-shielding efficiency as compared to the PVA/Lig-EA films with the same lignin mass content (Figure 5). It is worth noting that the PVA/Lig-OA and PVA/Lig-LA films with a lignin mass content of 4% block UV radiation in the UVA range by 96% and 87%, respectively, and completely block UV radiation in the UVB range. However, the increase in the lignin content slightly worsened the visible light transparency.

Thus, the lignin obtained as a by-product can be used in composite polymer films to provide effective UV shielding.

## 3. Materials and Methods

### 3.1. Materials

Mechanically crushed common reed straw (*Phragmites australis*) (Astrakhan region, Russia) was used in the study. All commercially available chemicals were of high purity and were used without further purification, namely, 2,2′-azino-bis(3-ethylbenzothiazoline-6-sulfonic acid) diammonium salt (ABTS), potassium persulfate, 1,1-diphenyl-2-picrylhydrazyl radical (DPPH^•^), (6-hydroxy-2,5,7,8-tetramethychroman-2-carboxylic acid (Trolox 97%), tetrahydrofuran (THF), DMSO-D_6_, Folin–Ciocâlteu reagent, and gallic acid (Sigma-Aldrich, Saint Louis, MO, USA), dimethylsulfoxide (DMSO, Marbiopharm, Yoshkar-Ola, Russia), ethanolamine (Component-Reaktiv, Moscow, Russia), and choline chloride, lactic acid (90%), oxalic acid, and poly(vinyl alcohol) (Mw ~ 86,000) (Acros Organics, Geel, Belgium).

All the solutions were prepared using water purified with a Simplicity^®^ Water Purification System (Merck KGaA, Darmstadt, Germany).

### 3.2. DESs Synthesis, Reed Straw Pretreatment, and Lignin Recovery

All the DESs, choline chloride/oxalic acid (molar ratio 1:1), choline chloride/lactic acid (molar ratio 1:5), and choline chloride/monoethanolamine (molar ratio 1:6), were obtained using a thermal mixing procedure.

The conditions for the reed straw pretreatment with DESs were selected based on the highest yield of reducing sugars and glucose after enzymatic treatment of the cellulosic substrate [36,37]. In brief, a mixture containing 20 g of crushed reed straw and 380 g of DES (loading 5 wt.%) was heated to 80 °C and incubated at this temperature and at constant stirring (8 h when using choline chloride/oxalic acid and 24 h when using choline chloride/lactic acid and choline chloride/monoethanolamine). Then, 400 mL of ethanol was added to the reaction mixture, and the cellulosic substrate was separated by vacuum filtration. The amount of 3.6 L of deionized water was added to the liquid fraction containing lignin and incubated for 24 h at room temperature. The lignin precipitate was separated by vacuum filtration, washed repeatedly with deionized water (to neutral pH), and dried at 60 °C to constant weight.

### 3.3. Preparation of Nanosized Lignin Particles (NPLig)

Nanosized lignin particles were obtained following a procedure from [64]. Lignins were dissolved in DMSO at a concentration of 10 mg mL^−1^, filtered through a syringe filter with a pore size of 0.45 μm, and placed in a dialysis bag (dialysis membrane Membra-Cel™ 14 kDa, Carl Roth, Karlsruhe, Germany), which was then immersed in an excess of periodically replaced deionized water. The dialysis process was performed for at least 24 h with stirring. After this, aqueous lignin suspensions were subjected to sonication for 1 h and centrifuged (1500× *g*, 20 min), and the supernatant containing nanolignin was separated. Finally, the resulting nanolignin dispersions were stored at 22 °C for further analysis. The average size and zeta potential of the lignin particle dispersions were measured using a Photocor Complex correlation spectrometer and a Photocor Compact-Z instrument (Photocor Instruments, Inc., College Park, MD, USA).

### 3.4. Preparation of Lig/PVA Composite Films

The composite films were prepared using a facile solution-casting method. PVA was dissolved in 90 °C deionized water and cooled to room temperature to form a transparent solution with a concentration of 5.0 wt.%. Then 80, 40, 20, and 10 μL of lignin solution in DMSO (concentration 25 mg mL^−1^) were added to 1 mL of PVA solution. Thus, the weight of the lignins was 4, 2, 1, and 0.5% of the weight of the PVA, respectively. Each mixture was vigorously mixed, poured onto a degreased glass plate to air-dry overnight, and then oven-dried at 50 °C for 24 h. The resulting composite films were quite uniform, with an average thickness of about 60 μm. After drying, the films were peeled from the glass plate and stored in desiccators at room temperature. The UV-shielding performance and optical transparency of the composite films were measured using a Shimadzu UV1240 mini spectrophotometer (Shimadzu Europa GmbH, Duisburg, Germany).

### 3.5. Characterization of Lignins

The weight-averaged molecular weight and the polydispersity index of the lignin samples were determined by gel penetration chromatography (Waters, Milford, MA, USA) using a UV detector. Previously, the lignin samples were derivatized by acetylation in an acetic anhydride/pyridine solution [73] and dissolved in THF. Standard polystyrene samples were used to construct a calibration curve.

The contents of polysaccharides in the lignin samples were analyzed according to the literature [47]. The total phenolic content (TPC) of lignin samples was determined by the Folin–Ciocâlteu spectrophotometric method using DMSO as a solvent and gallic acid as a reference compound [60]. The assay was done in triplicate, and TPCs were expressed as mg of gallic acid equivalents (GAE) per g of the dried sample.

The absorption spectra of the lignin samples in DMSO were recorded with a Shimadzu UV 1240 mini spectrophotometer (Shimadzu Europa GmbH, Duisburg, Germany) in a quartz cuvette with an optical path length of 1 cm. ATR-FTIR spectra were recorded on a Spectrum Two™ FT-IR spectrometer (PerkinElmer Inc., Waltham, MA, USA). ^1^H and ^13^C NMR spectra, including the J-modulated spin-echo-^13^C NMR spectra of the lignin samples, were recorded in DMSO-D_6_ at 303 K on an NMR spectrometer, the Bruker AVANCE 600 (Bruker, Karlsruhe, Germany) with an operating frequency of 600.03 MHz for ^1^H nuclei using techniques described in [74].

### 3.6. Determination of Antioxidant Activity

The analysis of DPPH^•^ and ABTS^+•^ scavenging was carried out using a spectrophotometric method on a Synergy 2 microplate photometer–fluorometer (BioTek, Winooski, VT, USA). DMSO was used to dissolve the lignin samples at various concentrations. The commercial antioxidant Trolox was used as a positive control.

The DPPH^•^ scavenging activity was determined according to [75] with some modifications. Eight mg of DPPH^•^ was dissolved in 100 mL of ethanol and then diluted with ethanol to obtain an optical density of 1.5 ± 0.02 at 517 nm. One hundred μL of the diluted DPPH^•^ solution and one hundred μL of the test sample solution at various concentrations were mixed in a microplate well, and the decrease in optical density was measured at 517 nm. The absorbance of the reaction mixture after 30 min was used to calculate the radical scavenging activity for each concentration. The blank sample consisted of 100 μL DMSO and 100 μL of the DPPH^•^ solution.

ABTS^+•^ scavenging activity was determined as described in [76] with some modifications. First, a stock solution of ABTS^+•^ was prepared (7 mM ABTS and 2.45 mM potassium persulfate in deionized water) and incubated in the dark at room temperature for 16 h. Before use, the stock solution was diluted with deionized water to obtain an optical density of 0.7 ± 0.02 at 734 nm. One hundred eighty μL of the diluted ABTS^+•^ solution and twenty μL of the test sample were mixed in a microplate well at various concentrations. The absorbance of the reaction mixture after 6 min was used to calculate the radical scavenging activity for each concentration. The blank sample consisted of 20 μL DMSO and 180 μL of the ABTS^+•^ solution.

All the measurements were carried out in triplicate. The radical scavenging activity (RSA) in DPPH^•^ and ABTS^+•^ assays was calculated according to the following formula:

$$RSA(\%)=\frac{A_{0}-A_{i}}{A_{0}}*100,$$

where *A*_0_ was the absorbance of the blank sample and *A_i_* was the absorbance in the presence of the test compound at different concentrations. The IC_50_ value was the sample concentration required for 50% scavenging of radicals, which was calculated from the curve of the RSA with the concentration.

## 4. Conclusions

A comparative study of the lignins obtained as by-products after treating reed straw with DESs of various compositions under conditions corresponding to maximum enzymatic hydrolysis of the cellulose substrate was performed. The low content of polysaccharides (3.4–3.9 wt.%) in the lignin samples enables their use without additional processing in order to obtain various high-value-added products. The entire set of experiments performed in this work showed that the composition of DESs has a significant (if not the main) effect on the structure and, therefore, on the properties of extracted lignin. The lignin obtained after treating reed straw with DESs based on choline chloride and oxalic acid (molar ratio 1:1) had the highest antioxidant activity and best UV-shielding properties. The IC_50_ values of Lig-OA were 10 and 50 μg mL^−1^ in the ABTS^+•^ and DPPH^•^ assays, and the PVA/Lig-OA films with a lignin mass content of more than 2% effectively blocked UV radiation. The high antioxidant activity of the lignin samples, and especially of Lig-OA, suggests that they can act as potential antioxidants, and transparent Lig/PVA films with good UV-shielding performance are compelling for food and drug packaging.

## Figures and Tables

**Table 1:** Phenolic content and antioxidant activity (IC_50_ value) of lignin samples.

Sample	TPC ^1^	ABTS ^2^	DPPH ^2^
Lig-OA	169.67 ± 1.45	10	50
Lig-LA	77.17 ± 1.48	30	200
Lig-EA	23.11 ± 0.73	60	500
Trolox	–	3	10

^1^ Total phenolic content (TPC) expressed as concentration of polyphenol (mg) in terms of the gallic acid equivalent (GAE) per g of lignin samples. ^2^ The concentration of the sample (μg mL^−1^) that can scavenge 50% radicals.

## Data Availability

Data is contained within the article and Supplementary Materials.

## Supplementary Materials

The following supporting information can be downloaded at https://www.mdpi.com/article/10.3390/ijms25158277/s1.
