# Supplementary material for: Green Extraction of Reed Lignin: The Effect of the Deep Eutectic Solvent Composition on the UV-Shielding and Antioxidant Properties of Lignin
Source: Int J Mol Sci. 2024 Jul 29;25(15):8277. doi: 10.3390/ijms25158277 (PMC11312954; doi:10.3390/ijms25158277)
Supplement: Supplementary file 1 [file ijms-25-08277-s001.zip › ijms-3097327-supplementary.pdf]

Supplementary Materials

Green extraction of reed lignin: the effect of the deep eutectic solvent composition on UV-shielding and antioxidant properties of lignin

Olga Morozova [1], Irina Vasil'eva [1], Galina Shumakovich [1], Maria Khlupova [1], Vyacheslav Chertkov [2], Alla Shestakova [3] and Alexander Yaropolov [1,*]

[1] A. N. Bach Institute of Biochemistry, Research Center of Biotechnology of the Russian Academy of Sciences, Leninsky Ave. 33, 119071 Moscow, Russia; morozova@inbi.ras.ru (O.M.); ir-vas@yandex.ru (I.V.); shumakovich1945@yandex.ru (G.S.); dave80@yandex.ru (M.K.)
[2] Department of Chemistry, Lomonosov Moscow State University, Leninskie Gory 1/3, 119991 Moscow, Russia; vchertkov@hotmail.com
[3] State Research Institute of Chemistry and Technology of Organoelement Compounds, Shosse Entuziastov 38, 111123 Moscow, Russia; alshestakova@yandex.ru
* Correspondence: yaropolov@inbi.ras.ru or alexander-yaropolov52@yandex.ru

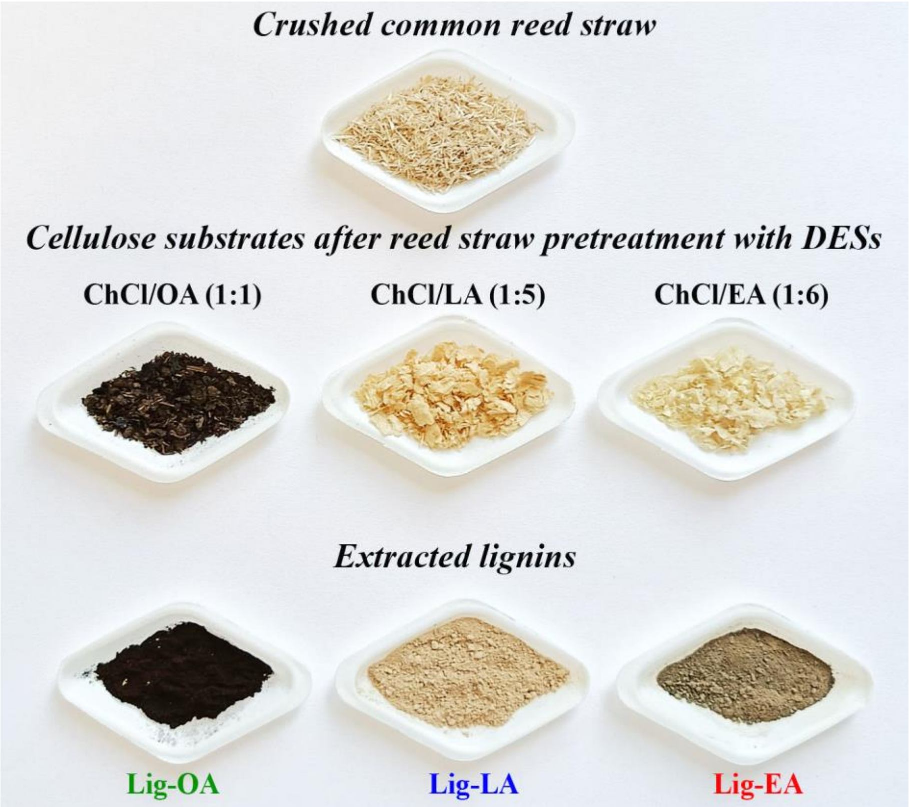

Figure S1. Digital photographs of crushed common reed straw, cellulose substrates after DES pretreatment and extracted lignins.

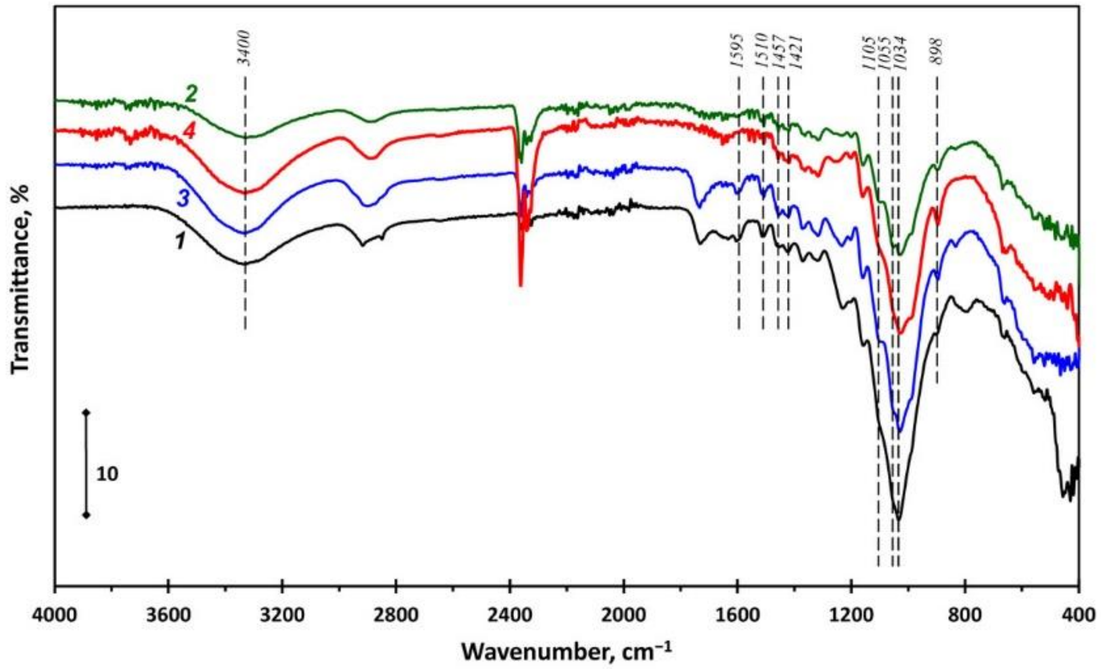

Figure S2. The ATR-FTIR spectra of reed straw (*1*) and cellulose substrates after reed straw pretreatment with various DESs: ChCl/OA (*2*), ChCl/LA (*3*) and ChCl/EA (*4*).

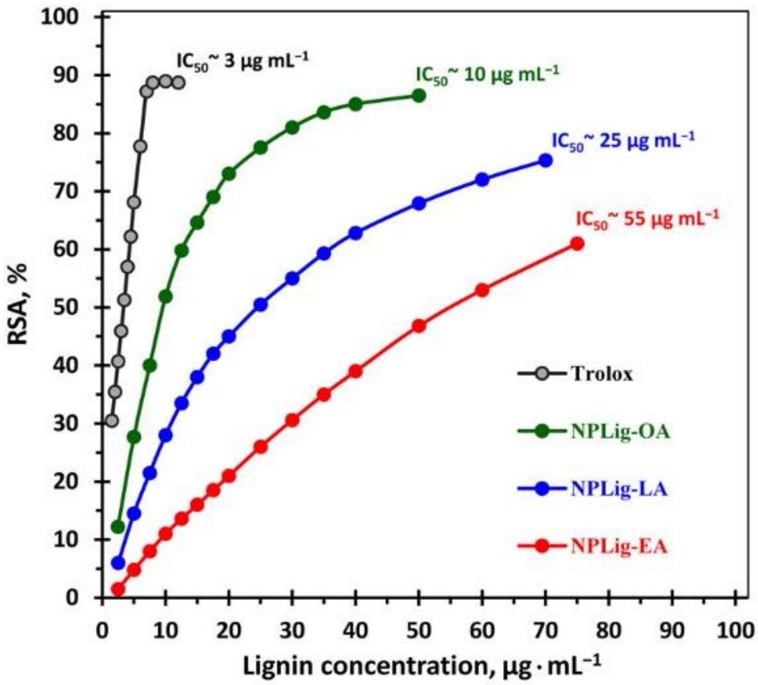

Figure S3. $ABTS^{\bullet+}$ radical scavenging activity of nanosized lignin particles NPLig-OA, NPLig-LA, NPLig-EA in comparison to Trolox.
